# Human oocytes and zygotes are ready for ultra-fast vitrification after 2 minutes of exposure to standard CPA solutions

**DOI:** 10.1038/s41598-019-52014-x

**Published:** 2019-11-05

**Authors:** Miguel Gallardo, Jaime Saenz, Ramon Risco

**Affiliations:** 1 Seville Engineering School, University of Seville, Camino de los Descubrimientos s/n, 41092 Seville, Spain; 2 Clínicas Ginemed, Calle Farmacéutico Murillo Herrera, 3, 41010 Seville, Spain; 3 National Accelerators Centre, Calle Thomas Alva Edison, 7, 41092 Seville, Spain

**Keywords:** Biophysical methods, Physics

## Abstract

Vitrification of human oocytes and embryos in different stages of development is a key element of daily clinical practice of *in vitro* fertilization treatments. Despite the cooling and warming of the cells is ultra-fast, the procedure as a whole is time consuming. Most of the duration is employed in a long (8–15 minutes), gradual or direct exposure to a non-vitrifying cryoprotectant solution, which is followed by a short exposure to a more concentrated vitrifying solution. A reduction in the duration of the protocols is desirable to improve the workflow in the IVF setting and reduce the time of exposure to suboptimal temperature and osmolarity, as well as potentially toxic cryoprotectants. In this work it is shown that this reduction is feasible. *In silico* (MatLab program using two-parameter permeability model) and *in vitro* observations of the oocytes’ osmotic behaviour indicate that the dehydration upon exposure to standard cryoprotectant solutions occurs very fast: the point of minimum volume of the shrink-swell curve is reached within 60 seconds. At that point, intracellular water ejection is complete, which coupled with the permeation of low molecular weight cryoprotectants results in similar intracellular and extracellular solute concentrations. This shows that prolonging the exposure to the cryoprotectant solutions does not improve the cytosolic glass forming tendency and could be avoided. To test this finding, human oocytes and zygotes that were donated for research were subjected to a shortened, dehydration-based protocol, consisting of two consecutive exposures of one-minute to two standard cryoprotectant solutions, containing ethylene glycol, dimethyl sulfoxide and sucrose. At the end of this two-minute dehydration protocol, the critical intracellular solute concentration necessary for successful vitrification was attained, confirmed by the post-warming survival and ability to resume cytokinesis of the cells. Further studies of the developmental competency of oocytes and embryos would be necessary to determine the suitability of this specific dehydration protocol for clinical practice, but based on our results, short times of exposure to increasingly hypertonic solutions could be a more time-efficient strategy to prepare human oocytes and embryos for vitrification.

## Introduction

The vitrification technique is a key element of *in vitro* fertilization units’ daily clinical practice: over one hundred-thousand embryos and thirty-thousand oocytes originated in human assisted reproduction treatments were cryopreserved in Spain in 2015^1,2^. The procedure relies on performing the cooling of the cells, and most importantly, the warming, at such high rates that the cytosol -and the extracellular solution in which the cell(s) are suspended- undergoes a second order phase transition to become an amorphous glass^3^. The water molecules in the cytosol do not have the necessary time, as temperature drops to a stable cryogenic range during cooling, and when it returns to room temperature during warming, to rearrange into ice crystals and grow to a size that can produce cellular injury or death. Instead, molecules remain in a similar disposition to the liquid state, but acquire the properties of a solid^4^. This liquid to solid transition, the glass transition, allows the cells’ structure and function to be preserved^5–7^. The chances of vitrification depend mostly on the speed at which cooling and warming occur, and the glass forming tendency of the solution, determined at large by its viscosity and solute concentration^8,9^.

In the human embryology laboratory, oocytes and embryos are cryopreserved in vitrification carriers that, according to their design, will have variable thermal exchange rates^10^. The cytosol of oocytes and embryos’ blastomeres, due to their large size and water content, does not vitrify at the rates currently attainable by those vitrification carriers, unlike spermatozoa^11^. For that reason, the cells are subjected to a preparation protocol prior to cooling, in which they lose water and are permeated by low molecular weight cryoprotectants (CPAs), increasing the cytosol’s solute concentration, and hence its glass forming tendency^8^. As a result, the cooling and warming rates necessary to avoid deleterious ice formation are lower. Irrespectively of the vitrification carrier of choice, most preparation protocols to ready oocytes and embryos for vitrification are very similar^12^. They usually feature two or more exposure phases —gradual or direct— to increasingly hypertonic solutions composed of permeating and non-permeating CPAs. The composition of these solutions and the duration of the cell’s exposure to them is critical, as CPA toxicity is both time and concentration dependent^13^.

Despite shorter protocols have already been proposed^9,14,15^, most of currently employed protocols^16–18^ feature an initial long-exposure phase lasting from 8 to 15 minutes to a non-vitrifying solution: a solution with a concentration of solutes that would not vitrify if cooled at the rates achievable with a specific carrier^19^. When exposed to hypertonic solutions, cells initially lose water and shrink to a minimum volume very rapidly. After that point, they slowly swell back towards their isotonic volume, as intra- and extracellular solute concentrations tend to osmotic equilibrium^20^. For this reason, this phase is usually termed equilibration. This is followed by a short exposure to a more concentrated solution, that vitrifies upon cooling at high rates (vitrification solution, VS)^21^. It is in this solution that the cells are suspended, loaded in the carrier device, and cooled. This exposure to VS is short, and cells are not allowed to equilibrate; they shrink, further dehydrated and permeated by cryoprotectants. The duration of the exposure has to be carefully controlled and limited to 60 seconds to avoid excessive osmotic effect^8,22^.

The equilibration of the cells, complete or partial, with non-vitrifying solutions during their preparation for vitrification is what contributes to most of the procedure’s duration and makes current protocols time consuming. However, shorter protocols of preparation of embryos and oocytes for vitrification are feasible: the critical cytosolic solute content necessary for successful vitrification at currently attainable cooling and warming rates can be achieved faster. In this work, using *in silico* data from a biophysical permeability model of human oocytes and *in vitro* osmotic observations, we propose a short, dehydration-based protocol. Using a standard set of vitrification solutions, we limit the duration of the exposure to one minute, when cells are close to the minimum volume point of their shrink-swell curve. We analyse the molarity of the cytosol of the dehydration protocol compared to a standard protocol, and test its efficacy on discarded human oocytes and abnormally fertilized zygotes donated for research.

## Results

### *In silico* analysis of the equlibration and dehydration protocols of human M-II Oocytes

**Figure 1:**
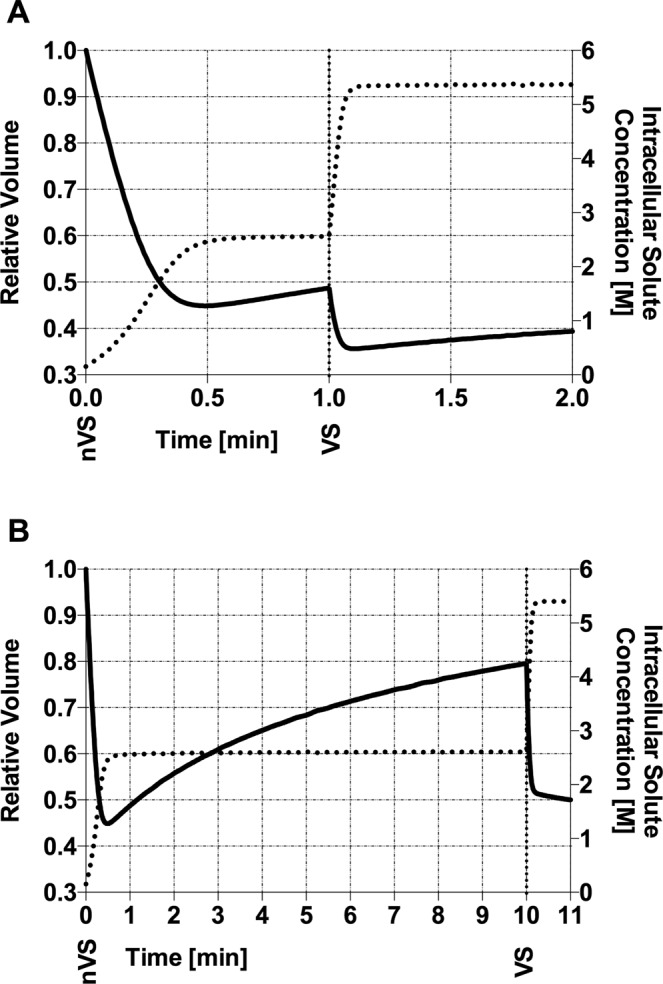
Results of the biophysical model, showing the history of relative volume and intracellular solute concentration of human M-II oocytes with A) DP and B) EP. Relative volume as solid line, intracellular solute concentration as dotted line. Exposure to nVS (nVS; 7.5% v/v Me_2_SO and 7.5% v/v EG) starts at t = 0, and the change to VS (VS; 15% v/v Me_2_SO, 15% v/v EG and 0,5 M sacarose) is demarcated in the X-axis with a vertical dotted line. Relative volume equals cell volume by the initial cell volume. Intracellular solute concentration in moles of solutes per liter.

The predicted relative volume and total intracellular solute concentration of oocytes subjected to two preparation protocols, the standard equilibration protocol (EP) and the short dehydration protocol (DP), as a function of time is shown on Fig. 1. Both protocols feature exposures to a non-vitrifying solution (nVS; 7.5% v/v Me_2_SO & 7.5% v/v EG; 1.06 mol/L Me_2_SO & 1.12 mol/L EG) and a vitrifying solution (VS; 15% v/v Me_2_SO, 15% v/v EG and 0,5 M sucrose; 2.11 mol/L Me_2_SO & 2.69 mol/L EG). In EP, the duration of exposure to nVS is of 10 minutes, and the duration of the exposure to VS is of 1 minute. In DP, the duration of the exposure to both nVS and VS is of 1 minute each.

In both protocols, model predictions show that after exposure to nVS, oocytes experiment a fast dehydration, shrinking to a minimum of 44.8% of their isotonic volume after 29.4 seconds of exposure. At the end of the exposure to nVS in DP, after 1 minute, equilibration has barely started, with the volume increasing to 48.6%. In the EP, exposure to nVS is prolonged to 10 minutes to allow for equilibration, and the oocyte furtherly swells back to 79.4% of its isotonic volume. Intracellular osmolarity, however, is very similar after either 1 or 10 minutes of exposure to nVS (Table 1 & Fig. 1).

**Table 1:** Key parameters that influence the survival of the oocyte to the vitrification process.

		(a)	(b)	(c)	(d)	(e)
		Duration of exposure	Intracellular Molarity	Permeated CPAs’ contribution to total intracellular molarity	$\frac{{\bf{Cell}}\,\,{\bf{Volume}}\,}{{\bf{Initial}}\,\,{\bf{Cell}}\,{\bf{Volume}}}$	$\frac{{\bf{Water}}\,{\bf{Volume}}\,}{{\bf{Initial}}\,{\bf{Active}}\,\,{\bf{Water}}\,{\bf{Volume}}}$
		(min)	mol/L	(%)	(%)	(%)
Equilibration Protocol	nVS	10	2.61	92.1	79.4	63.7
	VS	1	5.40	92.5	49.9	26.6
Dehydration Protocol	nVS	1	2.56	83.5	48.6	31.6
	VS	1	5.37	88.5	39.2	17.7

Comparison between equilibration and dehydration protocols of the results of the 2-P model for relevant osmotic parameters. (a) Time elapsed since oocytes are initially exposed to each solution; (b) Intracellular molarity of the oocyte; (c) Ratio of Intracellular mol/L of EG + DMSO by the total intracellular molarity; (d) Ratio of the oocyte’s volume by the initial isotonic total volume; (e) Water volume in the oocyte as a fraction of the initial active water volume of the oocyte. The molarity of nVS and VS is 2,41 mol/L and 5,30 mol/L, respectively.

Models show the oocyte subjected to the EP shrinking to 49.9% of its isotonic volume after 1 minute of exposure to the VS, whereas oocytes subjected to DP, with a previous shorter exposure to nVS, are predicted to shrink to 39.2%. Similar total intracellular molarity is achieved at the end of EP and DP, with 5.40 and 5.37 mol/L, respectively. However, in the EP, permeant cryoprotectants Me_2_SO and EG contribute to 92.5% of the intracellular molarity, vs. 88.5% in the DP protocol. The normalized water volume, on the other hand, is lower after DP compared to EP (17.7 vs. 26.6, respectively; Table 1).

### *In vitro* oocyte osmotic behaviour

The *in vitro* recordings of human oocytes exposed to EP and DP show the osmotic response of the oocytes to nVS and VS. The oocytes begun the experiment at an isosmotic condition of 290 mOsmol, and they were directly exposed to the hyperosmotic condition of nVS (2901 mOsmol). This osmotic gradient resulted in a volumetric excursion: the minimum relative volume attained was 60% of the oocyte’s initial volume in nVS; it took slightly longer than predicted to do so, from 40 to 60 seconds in both groups, likely due to the slight dilution of the solution during manipulation. Oocytes subjected to EP recovered 77% of its initial volume after 10 minutes of exposure to nVS, while oocytes subjected to DP remained at 61% at the end of the 1 minute exposure to nVS.

Following the exposures of different duration to nVS, the oocytes in both groups were exposed for 1 minute to the more concentrated VS (7068 mOsmol). The hyperosmotic conditions again provoke the shrinking of the cells, to 67% in the case of EP oocytes and to 52% of their initial volume in DP oocytes.

### Survival and viability of human oocytes and zygotes to vitrification and warming after the short dehydration protocol

As shown in Table 2, all of the oocytes and zygotes subjected to the experimental vitrification protocol DP were positive for survival (30/30 and 27/27, respectively). The observed survival of the oocytes was compared to the expected benchmark outcome of 95% survival in a normal population of oocytes, based on recent consensus^12^. Of 27 tripronuclear zygotes subjected to vitrification with DP, 24 resumed development and had cleaved after 24 hours of culture, compared to 25 of 27 fresh tripronuclear zygotes used as a control (p = 1).

**Table 2:** Survival and viability of human oocytes and zygotes after preparation for vitrification with the short protocol and vitrification with the SafeSpeed carrier.

No. of oocytes positive for survival after 2 hours/No. of oocytes subjected to vitrification	30/30 (100)
No. of zygotes positive for survival after 2 hours/No. tripronuclear zygotes subjected to vitrification	27/27 (100)
No. of cleaved embryos after 24 hours/No. tripronuclear zygotes subjected to vitrification	24/27 (88.8)
No. of cleaved embryos after 24 hours/No. of fresh tripronuclear zygotes	25/27 (92,6)

## Discussion

The survival and viability of unfertilized human oocytes and abnormally fertilized zygotes prepared with the dehydration protocol shows that their intracellular solute concentration was sufficiently high for the vitrification procedure to be successful at the cooling and warming rates obtained. This outcome was expected according to the 2-P model used to predict the osmotic behaviour of human M-II oocytes, as the intracellular molarity was similar after the standard EP, with 11 minutes of duration, and the DP, of 2 minutes. However, the exact composition of the cytosolic milieu, and hence its glass-forming tendency after each preparation protocol may differ. For instance, in DP, the contribution of permeating cryoprotectants Me_2_SO and EG to the final cytosolic solute concentration was slightly lower than in EP. On the other hand, EP resulted in lower intracellular free water content in the oocyte compared to EP. Relevant parameters that determine the glass-forming tendency of the cytosol, such as the viscosity of the milieu and the degree of hydrogen bonding between present water and intracellular macromolecules, could for this reason differ between oocytes prepared to vitrification with each protocol^22,23^. It should also be considered that we employed a carrier that is reported to produce higher rates of cooling and warming than others^15,24–30^ which may compensate for a possible lower intracellular glass forming tendency in the cells after DP^9,31^.

The protocols to prepare oocytes and embryos for vitrification do not only aim to achieve the appropriate intracellular glass-forming conditions, but aim to do so without impairing the ability of oocytes to fertilize and develop into viable embryos^12^. Several factors during preparation for vitrification can be detrimental, such as the type and concentration of cryoprotectants employed^8^, as well as the temperature^32,33^ and the duration of the exposure to these cryoprotectants^15,34^. Despite the tolerance of oocytes to these factors has been studied, reports regarding fertilization and developmental ability are less frequent due to ethical and legal constraints^35^. However, if we consider that several of the preparation protocols that are used daily in clinical practice resemble the EP described in this work^12,16–18^, it could be argued that current vitrification outcomes support the tolerance of the human M-II oocyte and embryo to the osmotic and mechanical stresses derived from the osmolarity of the nVS and VS employed (2901 and 7060 Osmolal, respectively). For instance, the effect on the meiotic spindle and oocyte competence of similar CPA exposure regimes, which according to the biophysical simulation produces shrinking to 50% of the oocyte’s isotonic volume, has been assessed on human^17^ and mice^26^ satisfactorily.

**Figure 2:**
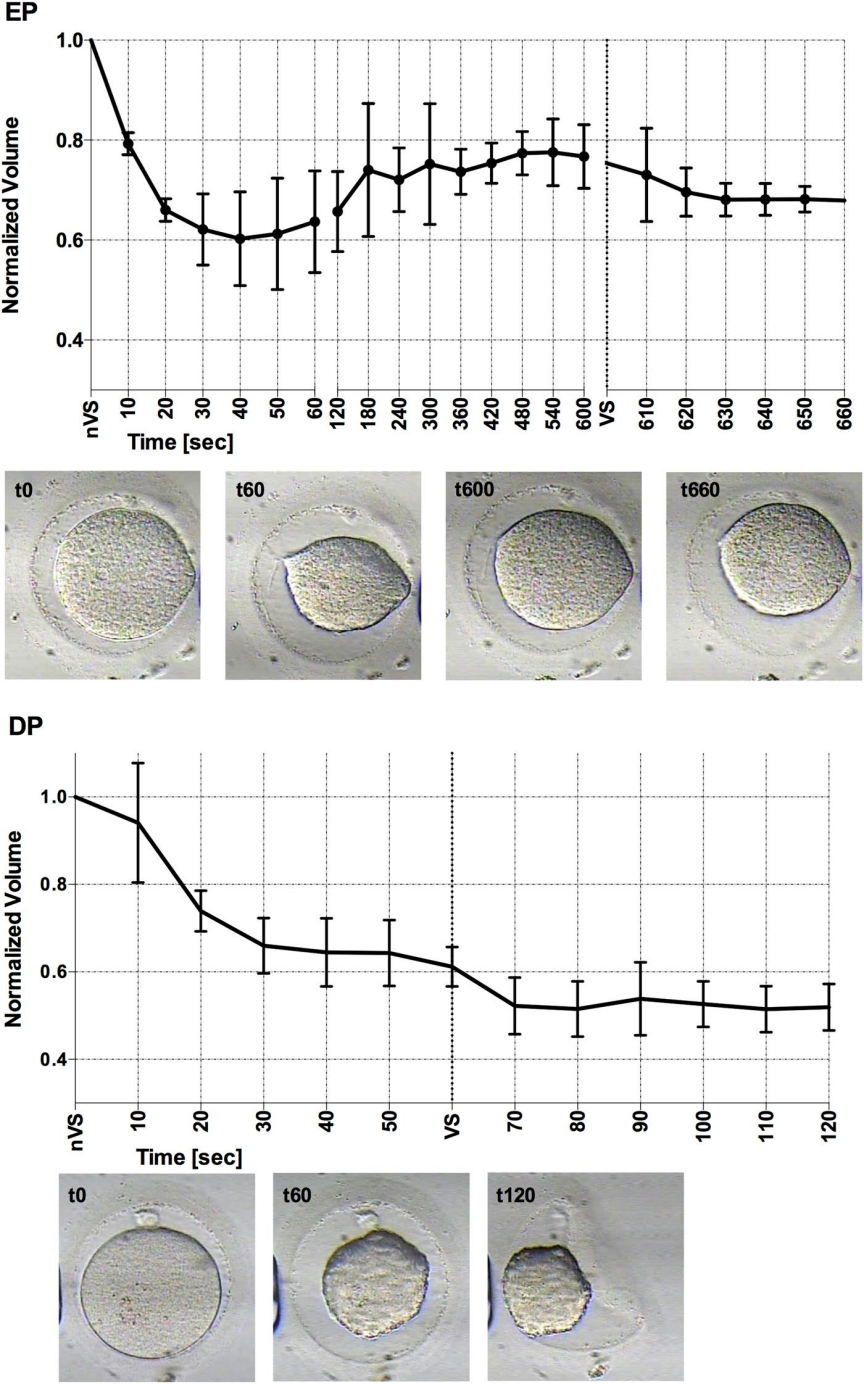
*In vitro* recording of the volumetric excursion of human M-II oocytes subjected to: Dehydration protocol (DP); Equilibration Protocol (EP) of preparation for vitrification. Exposure to nVS starts at t = 0, the change to VS is demarcated at the X-axis. An example of the oocyte’s morphology at relevant timeframes is shown below each graphic.

The osmotic gradient to which the oocytes are subjected in both protocols is very similar, as the final molarity after exposure to nVS is almost equivalent in EP and DP. However, the volumetric excursion cells undergo during preparation for vitrification are higher during DP: the oocyte is not allowed time to equilibrate and regain water and volume, and as a result, the total relative volume of the oocyte at the moment of cooling is lower, compared to a standard equilibration protocol (DP vs. EP, Fig. 2). Even if cell shrinkage is desirable, as it results in a more stable cytoplasm^8^, mechanical stress can compromise the developmental potential of oocytes, causing disruption in cytoskeleton structures, specially depolymerisation of the meiotic spindle^36–39^. In fact, the exact osmotic tolerance limit of oocytes is unclear^40^, and it has been conservatively estimated in previous attempts at optimizing preparation protocols^14,41–43^. As pointed out by Oda *et al*.^44^, fertilized mouse ova tolerate a contraction to 30% of their isotonic volume, concluding that shrinking alone is not likely to be the cause for viability impairment or death^44^. Also, certain strategies can be adopted to minimize the mechanical stress of shrinking-swelling^45^. Ultimately, the developmental ability of oocytes and embryos is the indicator of the safety and efficiency of a vitrification procedure^46^. Preliminary tests in the murine model would be necessary to confirm the validity of the proposed protocol for human oocyte and embryo cryopreservation^26,44^.

The *in silico* and *in vivo* analysis of the osmotic behaviour of the human oocyte are not without their limitations. When the relative volume of an oocyte is calculated from a bidimensional static frame-grab of the shrinking-swelling process, several assumptions are made, so the permeability parameters extrapolated from these observations might not be completely accurate^41,47^, and they were determined under lower concentrations of said CPAs^41,48–50^. At the higher concentrations employed, the cell could deviate from ideal osmotic behaviour^51^. At those concentrations, CPAs seem to interact with the cytoplasmic phospholipid bilayer and may alter its permeability^52^. Also, the biophysical parameters of oocytes seem to present high intervariability, potentially associated with many factors^43,53^. For these reasons, permeability models and *in vitro* observations might not reflect in a completely accurate fashion the biophysical reality of the process.

In spite of these limitations, we found a satisfactory agreement from our *in silico* and *in vitro* results, revealing that the point of minimum volume of the shrink-swell curve is attained within one minute of exposure to the hypertonic solutions employed^54^. This is relevant because, at this point, the intended osmotic effect of dehydration and CPA permeation was close to completion, with very similar intracellular and extracellular molarity. In consequence, limiting the exposure of the cells to the non-vitrifying and vitrifying hypertonic solutions to this point of minimum volume could be a more efficient approach to prepare them for vitrification, compared to the current approach consisting on a prolonged exposure to allow for partial osmotic equilibration with the non-vitrifying solution. Under this premise, we were able to successfully reduce the necessary time to prepare human oocytes and zygotes for vitrification to two minutes with a standard CPA combination. Similar time-efficient dehydration-based protocols comprising short exposures to increasingly hypertonic solutions could be fine-tuned for its application to other mammalian gametes and embryos, as well as more complex models.

## Materials and Methods

### Study population

Human Metaphase-II oocytes were chosen for the study as the need for their cryopreservation is rising due to the trend in social fertility preservation^55^. Also, there is room for improvements in the benchmark clinical outcomes as well as the procedure itself^12^.

In the interest of minimizing the number of biological material employed, previously characterized permeability parameters were used for the biophysical modelling^41,48,49,53^. With it, we theoretically identified a plausible, short dehydration protocol. Then, we validated the data by studying the *in vitro* osmotic behaviour of human M-II oocytes under the conditions used in the simulation. For these experiments, we employed a total of 22 Metaphase-II human oocytes retrieved in a private IVF setting by ovarian hyperstimulation, that had been discarded for clinical practice due to failed fertilization by ICSI and donated for research. For the subsequent viability assays of the dehydration protocol, we employed again discarded unfertilized human oocytes oocytes, and abnormally fertilized human zygotes, showing >2 pronuclei at the fertilization check 17 ± 2 hours post *in vitro* fertilization. These oocytes and zygotes were discarded from clinical practice and donated for research. In order to detect, with 80% power and alpha 0.05 a 15% one-sided difference from a benchmark survival rate to vitrification of 95%^12^, 30 oocytes and 27 zygotes were included in the study.

### Equilibration protocol and dehydration protocol to prepare M-II oocytes for vitrification

The Equilibration protocol (EP) was designed to represent the current standard practice to prepare human Metaphase-II oocytes for vitrification: the oocyte is directly exposed to a 7.5% v/v EG and 7.5% v/v Me_2_SO phosphate buffered saline (PBS) solution (non-vitrifying solution, nVS) for 10 minutes. This step is followed by a direct exposure to a 15% v/v EG, 15% v/v Me_2_SO and 0.5M sucrose PBS solution (vitrifying solution, VS) with 1 minute of duration. The Dehydration protocol (DP) is a modification of the Equilibration protocol, in which the exposure of the oocyte to both solutions nVS and VS is limited to 1 minute each.

### Cooling and warming to cryogenic temperatures in liquid nitrogen

For the viability experiments, oocytes and zygotes were prepared for vitrification following the short dehydration protocol (DP) and loaded in a closed vitrification carrier (Safepreservation, Spain), which was sealed hermetically in both ends prior to a fast plunging in liquid nitrogen. Note that the loading, sealing and cooling of the vitrification device were performed during the 60 seconds of exposure to VS, and not afterwards. The process of loading the vitrification carrier is described thoroughly elsewhere^24^.

For warming, the capillary was transferred to a 37 °C water bath, stirred for two seconds, dried in sterile cloth and its tip cut, releasing the oocytes in a 1M sucrose solution. After 60 seconds, oocytes were transferred to a 0.5M sucrose solution for three minutes and eventually placed for 5 minutes in a CPA-free solution. According to previous published measurements employing the same closed vitrification carrier, the zygotes were subjected to warming rates of 200.000 °C/min^24^.

### Modelling membrane permeability of the human M-II oocytes

#### The two-parameter transport formalism model

The two-parameter (2P) transport formalism has been used in this work to perform the necessary simulations to analyse the oocytes’ mass transfer dynamics in the presence of permeable and non-permeable solutes^20^. This model evaluates the flow of water and solutes through the cell membrane with a pair of coupled linear ordinary differential equations, assuming that there is no intermembrane interaction between water and the permeable solutes. Nevertheless, there are publications that have shown that the interaction factor or reflection coefficient (σ) of the 3-parameter Kedem-Katchalsky formalism is insignificant in many membranes, oocytes included^47,56^.

The 2-P model characterises the permeability of the membrane to water with the parameter Lp (hydraulic conductivity), and the permeability of the membrane to penetrant solutes with the parameter Ps, namely solute permeability. It is well known that permeability depends on temperature, and it is very common to define this relationship through an Arrhenius Eq. (1):

1 $$L={L}_{0}{e}^{\frac{-Ea}{R}(\frac{1}{Tref}-\frac{1}{T})}$$

the water transfer dynamics are defined by the Eq. (2)

2 $$\frac{dVw}{dt}=-\,{L}_{p}\cdot A\cdot R\cdot T\cdot ({M}^{e}-{M}^{i})$$

the permeable solute transfer dynamics is defined by the following Eq. (3):

3 $$\frac{dNs}{dt}=Ps\cdot A\cdot ({M}_{s}^{e}-{M}_{s}^{i})$$

In our particular case, we will need to define two solute equations, one for Me_2_SO and another for EG. This means that we will have to solve a system of three linear ordinary differential equations, with three parameters: Vw, *N*DMSO and *N*EG (Supplementary Table 1).

#### *In silico* analysis of the equlibration and dehydration protocols

In order to solve the system of differential equations, we developed specific software in MATLAB (Version R2014A, Mathworks, USA). In particular we employ the built-in function ODE45 based on the Runge-Kutta method.

The oocyte was assumed to be of perfect spherical shape, with a radius of 63 µm, and the osmotically inactive volume of the oocyte was considered to be 19% of its initial volume^48^. Values for the permeability of the cell membrane to water (hydraulic permeability; Lp), and to permeating cryoprotectants Me_2_SO and EG, and their respective activation energy (Ea), were used as previously estimated^41,49,57–59^ (Supplementary Table 2).

As in previous cell dynamic modelling studies, some simplifying assumptions were made: (i) the oocyte remained in perfect spherical shape during shrinking and swelling, with an area determined by the cell radius (A = 4 * π * r^2^); (ii) the solutions are ideal; (iii) hydrostatic pressure is zero; (iv) the osmotic coefficient for solutes are equal to 1, except for common salt (NaCl), with molalities and osmolalities equivalent and linear additivity of solute’s osmolalities. The cytosol was considered to contain only water and common salt (NaCl)^41,48,60^.

First, a simulation of a standard vitrification preparation protocol in which cell are left to equilibrate in the first solution (equilibration protocol, EP) was run. A second simulation was run with the shorter preparation protocol (dehydration protocol, DP), for which the exposure of the oocyte to both solutions nVS and VS is limited to 1 minute each. Key parameters for oocyte survival to vitrification, such as the volumetric excursion of the oocyte, intracellular active water content, and total cytosolic solute concentration were determined and compared between the two approaches.

### *In vitro* oocyte osmotic behaviour

To determine if the model predictions were accurate, the *in silico* dehydration profiles obtained with the theoretical models were reproduced with *in vitro* observations of the osmotic behaviour of human oocytes exposed to the standard and short preparation protocols.

#### Perfusion with CPA solutions

Drops of 2 µL of iso-osmotic PBS, 200 µL of nVS and 2 mL of VS were placed near contact in a 60 mm Petri dish. The Petri dish was placed on a heated stage at 25 °C (INUG2H-TIZSH, Tokai, Japan) on an inverted microscope (Ti-U, Nikon, Japan) with an assembled micromanipulator (Narishige, Japan). For each experiment, an oocyte was placed in the PBS drop with a capillary, and secured by suction of the zona pellucida by a holding pipette (MPH-MED-30, Origio, Denmark). To avoid desiccation, time between oocyte placement and perfusion of the solutions was minimized. The PBS drop and adjacent nVS drop were merged with the tip of the capillary to start the osmotic process. Once the exposure time to nVS was complete, the drop was merged with the adjacent VS drop. EP and DP protocols were replicated until we recorded five oocytes that retained a spherical morphology and presented fewer folds and creases during shrinking. Of a total of 22 oocytes subjected to the experiment, 5 replicates of each protocol were selected for analysis and calculation of the cytoplasmic area. Solutions were provided by SafePreservation (Spain).

#### Image acquisition and quantitative analysis of dehydration

The process was recorded in a PC (Software X) with a video acquisition system connected to the inverted microscope (Sony, Japan), using 400X magnification. A video frame of every ten seconds for the first minute of exposure on DS1 and DS2, and every 60 seconds after the first minute for the 10 minute exposure to DS1 was extracted and analyzed to determine the volumetric excursion of the oocyte relative to the initial isotonic volume. Still images of the oocytes were processed to determine their area for volume calculation, as described by^41^. To reduce skewing by irregularities in the spherical shape of the oocyte during shrinking, the radius was calculated from the oocyte’s bidimensional area, and then used to determine the oocyte’s volume, assuming a perfect spherical shape maintained throughout the process.

### Survival and viability of human oocytes and zygotes to vitrification and warming after the short dehydration protocol

The survival of the oocytes and zygotes was assessed two hours after completing the warming and rehydration process: those whose morphological appearance remained similar to fresh after vitrification were considered positive for survival^12^. As an additional viability indicator, post-warming mitosis resumption and cleavage was assessed after 24 hours of culture. Results were compared with a control group of 27 abnormal zygotes not subjected to vitrification. Embryo culture was carried out in pre-equilibrated embryotested 60 mm petri dishes (Falcon, Corning, USA), containing 30 µl drops of G1-Plus medium (Vitrolife, Sweden) with a mineral oil overlay at 37 °C and 6% CO_2_.

### Ethical approval and informed consent

The protocol of the study involving human material was reviewed and approved by a biomedical research ethics committee (CEI de los Hospitales Universitarios Virgen Macarena y Virgen del Rocío; Internal code 1123-M1-17). Informed consent was obtained from all participants and the protocol was in accordance with national guidelines and regulations.

## Supplementary information

Supplementary Material

## Data Availability

The data that support the findings of this study are available from the corresponding author, R.R., or the first author, M.G., upon reasonable request.
