# Supplementary material for: Human oocytes and zygotes are ready for ultra-fast vitrification after 2 minutes of exposure to standard CPA solutions
Source: Sci Rep. 2019 Nov 5;9:15986. doi: 10.1038/s41598-019-52014-x (PMC6831692; doi:10.1038/s41598-019-52014-x)
Supplement: Supplementary file 1 — Supplementary Material [file 41598_2019_52014_MOESM1_ESM.docx]

**Human oocytes and zygotes are ready for ultra-fast vitrification after 2 minutes of exposure to standard CPA solutions**

*Miguel Gallardo^1, 2^, Jaime Saenz^1^, Ramon Risco^1, 3, *^*

1. Seville Engineering School, University of Seville, Camino de los Descubrimientos s/n, 41092 Seville, Spain

2. Clínicas Ginemed, Calle Farmacéutico Murillo Herrera, 3, 41010, Seville, Spain.

3. National Accelerators Centre, Calle Thomas Alva Edison, 7, 41092 Seville, Spain

Correspondence and requests for materials should be addressed to R.R (ramon@us.es)

SUBJECT AREAS: BIOPHYSICS; EMBRIOLOGY; CRYOBIOLOGY; HUMAN ASSISTED REPRODUCTION

Keywords: Vitrification; cryobiology; human assisted reproduction; embryology.

**Supplementary Material**

Supplementary Table 1.

| Symbol | Meaning |
| --- | --- |
| w, s, sol, cpa | Subscripts (w, water; s, solutes; sol, cellular solutes; cpa, permeating cryoprotectants) |
| e, i, | superscripts; extracellular, intracellular |
| *d* | derivative of the function |
| V | Volume |
| A | Cell area |
| R | Universal gas constant |
| T | Absolute temperature |
| T_REF_ | Reference temperature |
| *t* | Time |
| *Lp* | Water permeability (hydraulic conductivity) |
| *L_O_* | Permeability at a reference temperature |
| *M* | Osmolality |
| *n* | Number of moles |
| $\overline{V}$ | Partial molar volume |
| *N* | Osmoles of solute |
| *P*s | Solute permeability |

Supplementary Table 1. Definition of symbols employed in equations 1-3.

Supplementary Table 2.

| Parameter | value; uds; 25 ºC; 1 atm | Reference. |
| --- | --- | --- |
| $Lp$ | 0,69 µm/min | (Hunter et al., 1992) |
| $E_{A}^{H2O}$ | 14,42 kcal/mol |  |
| $P_{Me2SO}$ | 15 µm/min | (Paynter, et al., 1999a) |
| $E_{A}^{Me2SO}$ | 23,52 kcal/mol |  |
| $P_{EG}$ | 9,16 µm/min | (Mullen et al., 2008) |
| $E_{A}^{EG}$ | 21,20 kcal/mol |  |
| $\bar{V}_{H20}$ | 18,61 cm^3^/mol | (Markarian, Asatryan, & Zatikyan, 2005). |
| $\bar{V}_{NaCl}$ | 16,68 cm^3^/mol | (Pitzer, Peiper, & Busey, 1984). |
| $\bar{V}_{Me2SO}$ | 64,323 cm^3^/mol | (Markarian, Asatryan, & Zatikyan, 2005). |
| $\bar{V}_{EG}$ | 55,31 cm^3^/mol | (Ambrosone et al., 1996). |

Supplementary Table 2. Relevant parameters for permeability modeling of human Metaphase-II oocytes.

Supplementary Material 3.

CODE FOR MATLAB SOFTWARE TO CALCULATE FLUX OF WATER, ETHYLENE GLYCOL AND DYMETHYL SULFOXIDE THROUGH THE PLASMA MEMBRANE OF THE HUMAN METAPHASE-II OOCYTE.

%*******************************************************************************

% Programa que permite calcular las dos etapas, primero la de deshidratación

% y luego la rehidratación con respecto al tiempo al añadir crioprotector.

%*******************************************************************************

r=63;%Radio del oocito[micras]

inactive=0.19;%Fraccion volumen celula osmoticamente inactivo

Vcelli=4/3*pi*r^3*1e-9;%volumen total inicial de oocito [mm3]

Vi=Vcelli*(1-inactive);%volumen inicial agua osmoticamente activo en oocito [mm3]

A=4*pi*r^2*1e-6;%area membrana celular oocito [mm2]

T=298.15;%temperatura a la que se añade el crioprotector [K]

MA=18.01528;%peso molecular del agua pura [g/mol]

VmA=18.61*1e-3;%volumen molar del agua pura a 25ºC [litros/mol]

roA=0.9970479 %Densidad del agua pura a 25ºC [Kg/litro]=[mg/mm3]

CM2=0.154;%concentracion isotonica inicial de ClNa intracelular[mol/litro]

v2=1.6836;%coeficiente de disociacion del ClNa a la concentracion isotonica

Vm2=16.68*1e-3;%volumen molar parcial a dilucion infinita de ClNa a 25ºC[litros/mol]

n2=CM2*Vi*1e-6;%moles iniciales de ClNa en el medio intracelular

nsol=n2*v2;%osmoles iniciales de de ClNa en el medio intracelular

Vm_sucrose=21.5412*1e-3;%volumen molar sacarosa [litros/osmol]=[cm3/mosmol]---PENDIENTE BUSCAR DATO REFERENCIADO---

Vm_EG=55.37*1e-3;%volumen molar parcial a dilucion infinita de EG a 25ºC[litros/osmol]=[cm3/mosmol]

Vm_DMSO=64.323*1e-3; %volumen molar parcial a dilucion infinita de DMSO a 25ºC[litros/osmol]=[cm3/mosmol]

%**************************************************************************

%DATOS DE PERMEABILIDAD DE LA MEMBRANA CELULAR A CPAs Y AGUA

%**************************************************************************

%Permeabilidad celular al agua---------------

Lpo=0.69;%Permeabilidad hidráulica del agua a 25ºC [um/min atm]

Ea_w=14.42;%Energia de activacion de la permeabilidad del agua [kcal/mol]

To_w=298.15;%Temperatura de referencia de la permeabilidad hidraúlica del agua [K]

Lp=Lpo*exp((-Ea_w*1e3/1.987207)*(1/T-1/To_w))*9.869232667;%Permeabilidad de la membrana al agua en presencia de EG [mm4/(min J)]

%Permeabilidad celular al CPA DMSO---------------

Po_DMSO=15;%Coeficiente de permeabilidad del DMSO a 24ºC[um/min]

Ea_DMSO=23.52;%Energia de activacion de la permeabilidad del DMSO [kcal/mol]

To_DMSO=297.15;%Temperatura de referencia de la permeabilidad DMSO [K]

P_DMSO=Po_DMSO*exp((-Ea_DMSO*1e3/1.987207)*(1/T-1/To_DMSO))*1e-3;%Permeabilidad de la membrana al DMSO [mm/min]

%Permeabilidad celular al CPA EG---------------

Po_EG=9.16;%Coeficiente de permeabilidad del EG a 25ºC[um/min]

Ea_EG=21.20;%Energia de activacion de la permeabilidad del EG [kcal/mol]

To_EG=298.15;%Temperatura de referencia de la permeabilidad EG [K]

P_EG=Po_EG*exp((-Ea_EG*1e3/1.987207)*(1/T-1/To_EG))*1e-3;%Permeabilidad de la membrana al EG [mm/min]

%##########################################################################

%##########################################################################

%ETAPA 1 ETAPA 1 ETAPA 1 ETAPA 1 ETAPA 1 ETAPA 1 ETAPA 1 ETAPA 1 ETAPA 1

%##########################################################################

%##########################################################################

%**************************************************************************

%CONCENTRACIONES DE CRIOPROTECTORES Y SACAROSA DEL MEDIO EXTRACELULAR

%**************************************************************************

CM_DMSO=1.056;%concentración molar de DMSO [osmol/litro]=[moslmol/cm3]

CM_EG=1.345;%concentración molar de Etilenglicol[osmol/litro]=[mosmol/cm3]

CM_sucrose=0;%concentración molar de sacarosa [osmol/litro]=[mosmol/cm3]

%**************************************************************************

%CALCULO MOLALIDADES Y FRACCIONES MOLARES DE LOS SOLUTOS EN EL MEDIO EXTRACELULAR

%**************************************************************************

nA=(1-CM2*Vm2-CM_DMSO*Vm_DMSO-CM_EG*Vm_EG-CM_sucrose*Vm_sucrose)/VmA;%moles de agua en un medio extracelular de un litro de volumen

masaA=nA*MA*1e-3;%Kg de agua(disolvente) en un litro de disolucion

%Osmolalidades en el medio extracelular----------------------

mB=v2*CM2/masaA;

m_DMSO=CM_DMSO/masaA;

m_EG=CM_EG/masaA;

m_sucrose=CM_sucrose/masaA;

Smex=mB+m_DMSO+m_EG+m_sucrose;%suma de las osmolalidades de los solutos del medio extracelular[osmol/kg]

%Fracciones molares en el medio extracelular---------------

ntot=CM2*v2+CM_DMSO+CM_EG+CM_sucrose+nA;%moles totales por litro de solucion extracelular[moles/litro]

XB=CM2/ntot;

X_DMSO=CM_DMSO/ntot;

X_EG=CM_EG/ntot;

X_sucrose=CM_sucrose/ntot;

XA=1-XB-X_DMSO-X_EG-X_sucrose;

%**************************************************************************

%DEFINICION DEL SISTEMA DE ECUACIONES DIFERENCIALES DE DESHIDRATACION

%**************************************************************************

%Las ecuaciones se escriben en un vector columna.

%la primera fila corresponde a la ec. de flujo de agua. El volumen de agua

%intracelular [mm3] es x(1)

%La segunda fila corresponde a la ec. de flujo de moles de CPA DMSO. Los

%mosmoles de CPA DMSO intracelular es x(2)

%La tercera fila corresponde a la ec. de flujo de moles de CPA EG. Los

%mosmoles de CPA intracelular es x(3)

R=8.314475;%[J/molK]

f=@(t,x) [-Lp*A*R*1e-3*T*(roA*Smex*1e-3-(nsol*1e3+x(2)+x(3))/x(1));P_DMSO*A*(roA*m_DMSO*1e-3-x(2)/x(1));P_EG*A*(roA*m_EG*1e-3-x(3)/x(1))];

%se asume una densidad del agua 1 es decir, que 1 litro=1Kg de agua.

%el factor 1e-3 que multiplica a R es para pasar los moles a mmoles(estan

%en el denominador)

%el factor 1e-3 que afecta a Smex es para pasar de osmol/Kg a mosmol/mg

%el factor 1e3 que afecta a nsol es para pasar de osmol a mosmol

%el factor 1e-3 que afecta a m_DMSO es para pasar de osmol/Kg a mosmol/mg

%el factor 1e-3 que afecta a m_EG es para pasar de osmol/Kg a mosmol/mg

%**************************************************************************

%INTEGRACION DEL SISTEMA DE ECUACIONES DIFERENCIALES QUE DEFINEN LOS FLUJOS

%DE AGUA [MM3] Y DE MOLES DE CPA [MOSMOL]

%**************************************************************************

tspan=[0:.01:1];%intervalo de tiempo en minutos

xo=zeros(1,3);

xo(1)=Vi;%El volumen de agua intracelular inicial es Vi[mm3]

xo(2)=0;%Los moles iniciales de CPA DMSO son cero dentro de la celula.

xo(3)=0;%Los moles iniciales de CPA EG son cero dentro de la celula.

[t,x]=ode45(f,tspan,xo);

%Resultados numericos-----------------------------------------------------

Vn=[];%inicializamos el vector de resultados con un vector nulo.

nfull=[];

Vn=(Vcelli-Vi+x(:,1)+x(:,2)*Vm_DMSO*1e3+x(:,3)*Vm_EG*1e3+nsol*1e3*Vm2*1e3)/Vcelli;%Fraccion del volumen celular total con respecto al inicial

nfull=x(:,2)+x(:,3)+nsol*1e3;%mosmoles intracelulares totales:DMSO+EG+ClNa

flux_w=Lp*A*R*1e-3*T*(Smex*1e-3-(nsol*1e3+x(:,2)+x(:,3))./x(:,1));%caudal de agua instantaneo a traves de la membrana [mm3/min]

flux_EG=P_EG*A*(m_EG*1e-3-x(:,3)./x(:,1));%caudal de mosmoles de Etilenglicol instantaneo a traves de la membrana [mosmol/min]

flux_DMSO=P_DMSO*A*(m_DMSO*1e-3-x(:,2)./x(:,1));%caudal de mosmoles de DMSO instantaneo a traves de la membrana [mosmol/min]

disp('VolumenRel minimo(%)///Tiempo(segundos)///Caudal inicial agua(mm3/min)///VolumenRel final(%)')

[minVol posVol]=min(Vn);

[minFlux posFlux]=min(flux_w);

minTime=t(posVol)*60;

[minVol*100 minTime flux_w(1) Vn(end)*100]

%Representacion grafica de los %resultados---------------------------------

f1=figure;

plot(t,Vn,'b')

hold on;

plot(t(posVol),minVol,'*r')

title('Evolucion deshidratacion celular: Ratio del volumen geometrico celular respecto al volumen total inicial')

ylabel('Fraccion volumen celular geometrico [adim]')

xlabel('Tiempo[min]')

f2=figure;

plot(t,flux_w,'b')

title('Tasa deshidratacion celular: Caudal de agua que atraviesa la membrana celular')

ylabel('Caudal volumentrico [mm3/min]')

xlabel('Tiempo[min]')

hold on;

f3=figure;

plot(t,flux_DMSO,'b',t,flux_EG,'g')

title('Tasa de entrada de crioprotector EG y DMSO al interior celular')

ylabel('Caudal molar [mosmol/min]')

xlabel('Tiempo[min]')

hold on;

f4=figure;%concentracion de solutos intracelulares

plot(t,x(:,2)./x(:,1),'b',t,x(:,3)./x(:,1),'g',t,nsol*1e3./x(:,1),'r',t,nfull./x(:,1),'k',t,Smex*1e-3,'*m')

title('Osmolalidad de crioprotector DMSO y EG en el interior celular')

ylabel('Osmolalidad [mosmol/mg]')

xlabel('Tiempo[min]')

hold on;

f5=figure;%fraccion de agua intracelular con respecto a la inicial

plot(t,x(:,1)/Vi*100)

title('Ratio del volumen de agua intracelular con respecto al agua inicialmente presente')

ylabel('Fraccion volumen agua intracelular [%]')

xlabel('Tiempo[min]')

hold on;

f6=figure;%concentracion de sal intracelular

plot(t,nsol*1e3./x(:,1),'r',t,mB*1e-3,'*k')

title('Osmolalidad de ClNa en el interior celular')

ylabel('Osmolalidad [mosmol/mg]')

xlabel('Tiempo[min]')

hold on;

f7=figure;%concentracion de DMSO intracelular

plot(t,x(:,2)./x(:,1),'b',t,m_DMSO*1e-3,'*k')

title('Osmolalidad de DMSO en el interior celular')

ylabel('Osmolalidad [mosmol/mg]')

xlabel('Tiempo[min]')

hold on;

f8=figure;%concentracion de EG intracelular

plot(t,x(:,3)./x(:,1),'g',t,m_EG*1e-3,'*k')

title('Osmolalidad de EG en el interior celular')

ylabel('Osmolalidad [mosmol/mg]')

xlabel('Tiempo[min]')

hold on;

%##########################################################################

%##########################################################################

%ETAPA 2 ETAPA 2 ETAPA 2 ETAPA 2 ETAPA 2 ETAPA 2 ETAPA 2 ETAPA 2 ETAPA 2

%##########################################################################

%##########################################################################

%**************************************************************************

%CONCENTRACIONES DE CRIOPROTECTORES Y SACAROSA DEL MEDIO EXTRACELULAR

%**************************************************************************

CM_DMSO=2.112;%concentración molar de DMSO [osmol/litro]=[moslmol/cm3]

CM_EG=2.69;%concentración molar de Etilenglicol[osmol/litro]=[mosmol/cm3]

CM_sucrose=0;%concentración molar de sacarosa [osmol/litro]=[mosmol/cm3]

%**************************************************************************

%CALCULO MOLALIDADES Y FRACCIONES MOLARES DE LOS SOLUTOS EN EL MEDIO EXTRACELULAR

%**************************************************************************

nA=(1-CM2*Vm2-CM_DMSO*Vm_DMSO-CM_EG*Vm_EG-CM_sucrose*Vm_sucrose)/VmA;%moles de agua en un medio extracelular de un litro [moles/litro]

masaA=nA*MA*1e-3;%[Kg/litro]

%Osmolalidades en el medio extracelular----------------------

mB=v2*CM2/masaA;

m_DMSO=CM_DMSO/masaA;

m_EG=CM_EG/masaA;

m_sucrose=CM_sucrose/masaA;

Smex=mB+m_DMSO+m_EG+m_sucrose;%suma de las osmolalidades de los solutos del medio extracelular[osmol/kg]

%Fracciones molares en el medio extracelular---------------

ntot=CM2*v2+CM_DMSO+CM_EG+CM_sucrose+nA;%moles totales por litro de solucion extracelular[moles/litro]

XB=CM2/ntot;

X_DMSO=CM_DMSO/ntot;

X_EG=CM_EG/ntot;

X_sucrose=CM_sucrose/ntot;

XA=1-XB-X_DMSO-X_EG-X_sucrose;

%**************************************************************************

%DEFINICION DEL SISTEMA DE ECUACIONES DIFERENCIALES DE DESHIDRATACION

%**************************************************************************

%Las ecuaciones se escriben en un vector columna.

%la primera fila corresponde a la ec. de flujo de agua. El volumen de agua

%intracelular [mm3] es x(1)

%La segunda fila corresponde a la ec. de flujo de moles de CPA DMSO. Los

%mosmoles de CPA DMSO intracelular es x(2)

%La tercera fila corresponde a la ec. de flujo de moles de CPA EG. Los

%mosmoles de CPA intracelular[mol] es x(3)

R=8.314475;%[J/molK]

f=@(t,x) [-Lp*A*R*1e-3*T*(roA*Smex*1e-3-(nsol*1e3+x(2)+x(3))/x(1));P_DMSO*A*(roA*m_DMSO*1e-3-x(2)/x(1));P_EG*A*(roA*m_EG*1e-3-x(3)/x(1))];

%se asume una densidad del agua de 1 litro=1Kg

%el factor 1e-3 que multiplica a R es para pasar los moles a mmoles

%el factor 1e-3 que afecta a Smex es para pasar de mol/litro a mosmol/mm3

%el factor 1e3 que afecta a nsol es para pasar de osmol a mosmol

%el factor 1e-3 que afecta a m_DMSO es para pasar de mol/litro a mosmol/mm3

%el factor 1e-3 que afecta a m_EG es para pasar de mol/litro a mosmol/mm3

%**************************************************************************

%INTEGRACION DEL SISTEMA DE ECUACIONES DIFERENCIALES QUE DEFINEN LOS FLUJOS

%DE AGUA [MM3] Y DE MOLES DE CPA [MOSMOL]

%**************************************************************************

tspan=[1:.01:2];%intervalo de tiempo en minutos

xo=zeros(1,3);

xo(1)=x(end,1);%Volumen de agua intracelular inicial es el final del anterior paso[mm3]

xo(2)=x(end,2);%Los osmoles iniciales de CPA DMSO dentro de la celula son los osmoles finales del paso anterior.

xo(3)=x(end,3);%Los osmoles iniciales de CPA EG dentro de la celula son los osmoles finales del paso anterior.

[t,x]=ode45(f,tspan,xo);

%Resultados numericos en una grafica---------------

Vn=[];

nfull=[];

Vn=(Vcelli-Vi+x(:,1)+x(:,2)*Vm_DMSO*1e3+x(:,3)*Vm_EG*1e3+nsol*1e3*Vm2*1e3)/Vcelli;%Fraccion del volumen celular total con respecto al inicial

nfull=x(:,2)+x(:,3)+nsol*1e3;%mosmoles intracelulares totales:DMSO+EG+ClNa

flux_w=Lp*A*R*1e-3*T*(Smex*1e-3-(nsol*1e3+x(:,2)+x(:,3))./x(:,1));%caudal de agua instantaneo a traves de la membrana [mm3/min]

flux_EG=P_EG*A*(m_EG*1e-3-x(:,3)./x(:,1));%caudal de mosmoles de Etilenglicol instantaneo a traves de la membrana [mosmol/min]

flux_DMSO=P_DMSO*A*(m_DMSO*1e-3-x(:,2)./x(:,1));%caudal de mosmoles de DMSO instantaneo a traves de la membrana [mosmol/min]

disp('VolumenRel minimo(%)///Tiempo minimo(s)///Caudal Inicial deshidrat(mm3/min)///VolumenRel Final(%)')

[minVol posVol]=min(Vn);

[minFlux posFlux]=min(flux_w);

minTime=t(posVol)*60;

[minVol*100 minTime flux_w(1) Vn(end)*100]

%Representacion grafica de los %resultados---------------------------------

figure(f1);

plot(t,Vn,'b')

hold on;

plot(t(posVol),minVol,'*r')

title('Evolucion deshidratacion celular: Ratio del volumen geometrico celular respecto al volumen total inicial')

ylabel('Fraccion volumen celular geometrico[adim]')

xlabel('Tiempo[min]')

grid on;

figure(f2);

plot(t,flux_w,'b')

title('Tasa deshidratacion celular: Caudal de agua que atraviesa la membrana celular')

ylabel('Caudal volumentrico [mm3/min]')

xlabel('Tiempo[min]')

grid on;

figure(f3);

plot(t,flux_DMSO,'b',t,flux_EG,'g')

title('Tasa de entrada de crioprotector DMSO y EG al interior celular')

ylabel('Caudal molar [mosmol/min]')

xlabel('Tiempo[min]')

grid on;

figure(f4);

plot(t,x(:,2)./x(:,1),'b',t,x(:,3)./x(:,1),'g',t,nsol*1e3./x(:,1),'r',t,nfull./x(:,1),'k',t,Smex*1e-3,'*m')

title('Osmolalidad de crioprotector DMSO,EG y sal en el interior celular')

ylabel('Osmolalidad [mosmol/mg]')

xlabel('Tiempo[min]')

grid on;

figure(f5);

plot(t,x(:,1)/Vi*100)

title('Ratio del volumen de agua intracelular con respecto al agua inicialmente presente')

ylabel('Fraccion volumen agua intracelular [%]')

xlabel('Tiempo[min]')

grid on;

figure(f6);%concentracion de sal intracelular

plot(t,nsol*1e3./x(:,1),'r',t,mB*1e-3,'*k')

title('Osmolalidad de ClNa en el interior celular')

ylabel('Osmolalidad [mosmol/mg]')

xlabel('Tiempo[min]')

grid on;

figure(f7);%concentracion de DMSO intracelular

plot(t,x(:,2)./x(:,1),'b',t,m_DMSO*1e-3,'*k')

title('Osmolalidad de DMSO en el interior celular')

ylabel('Osmolalidad [mosmol/mg]')

xlabel('Tiempo[min]')

grid on;

figure(f8);%concentracion de EG intracelular

plot(t,x(:,3)./x(:,1),'g',t,m_EG*1e-3,'*k')

title('Osmolalidad de EG en el interior celular')

ylabel('Osmolalidad [mosmol/mg]')

xlabel('Tiempo[min]')

grid on;
